# Supplementary material for: Inflammatory factors and risk of lung adenocarcinoma: a Mendelian randomization study mediated by blood metabolites
Source: Front Endocrinol (Lausanne). 2024 Aug 27;15:1446863. doi: 10.3389/fendo.2024.1446863 (PMC11384989; doi:10.3389/fendo.2024.1446863)
Supplement: Supplementary file 1 [file DataSheet1.zip › supplementary files/S2/GCST90200535/sensitivity-analysis.pdf]

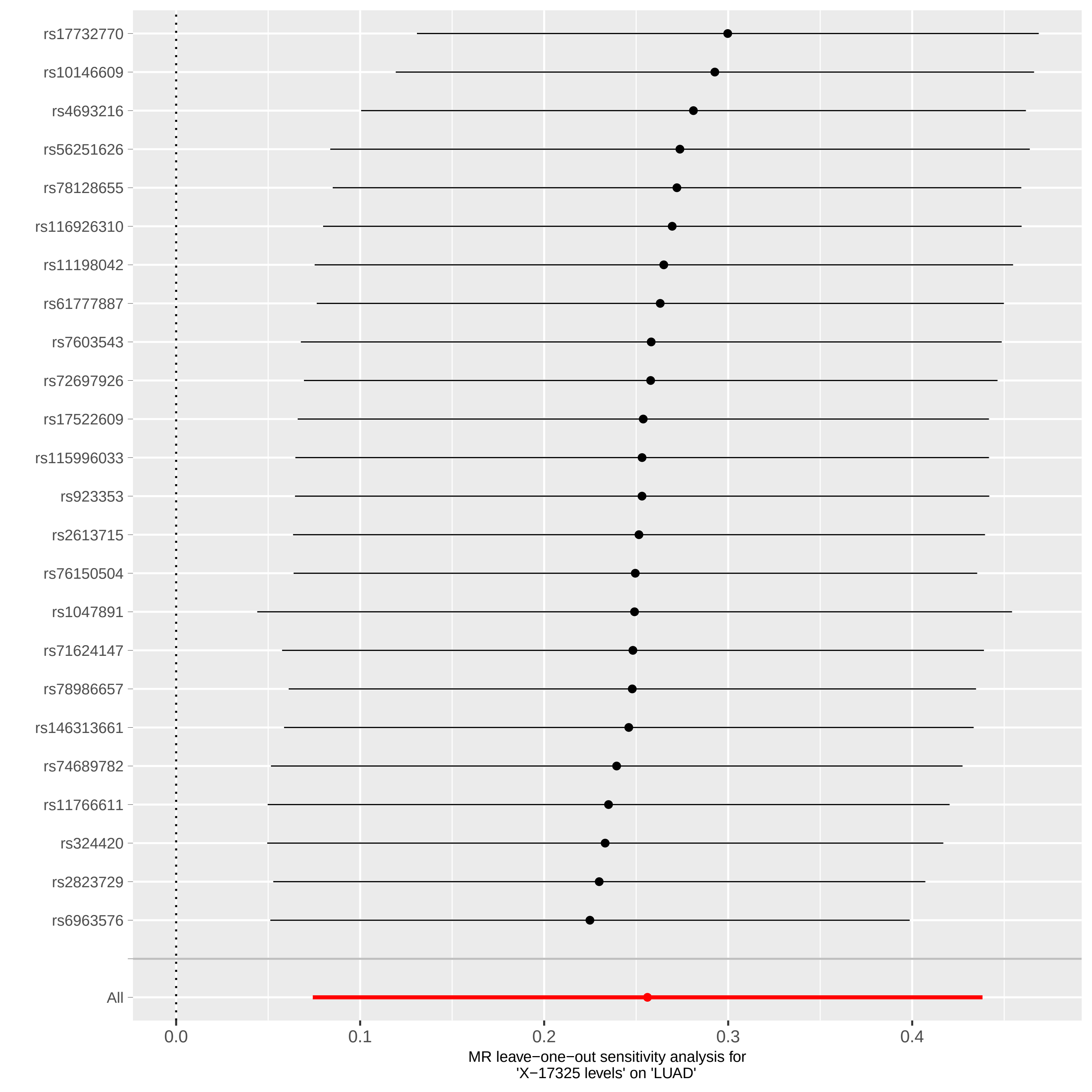

rs17732770
rs10146609
rs4693216
rs56251626
rs78128655
rs116926310
rs11198042
rs61777887
rs7603543
rs72697926
rs17522609
rs115996033
rs923353
rs2613715
rs76150504
rs1047891
rs71624147
rs78986657
rs146313661
rs74689782
rs11766611
rs324420
rs2823729
rs6963576
All
0.0
0.1
0.2
0.3
0.4
MR leave−one−out sensitivity analysis for
'X−17325 levels' on 'LUAD'
